# Supplementary figures and images for: Bioaccumulation of PCBs and PBDEs in Fish from a Tropical Lake Chapala, Mexico
Source: Toxics. 2021 Sep 29;9(10):241. doi: 10.3390/toxics9100241 (PMC8540629; doi:10.3390/toxics9100241)

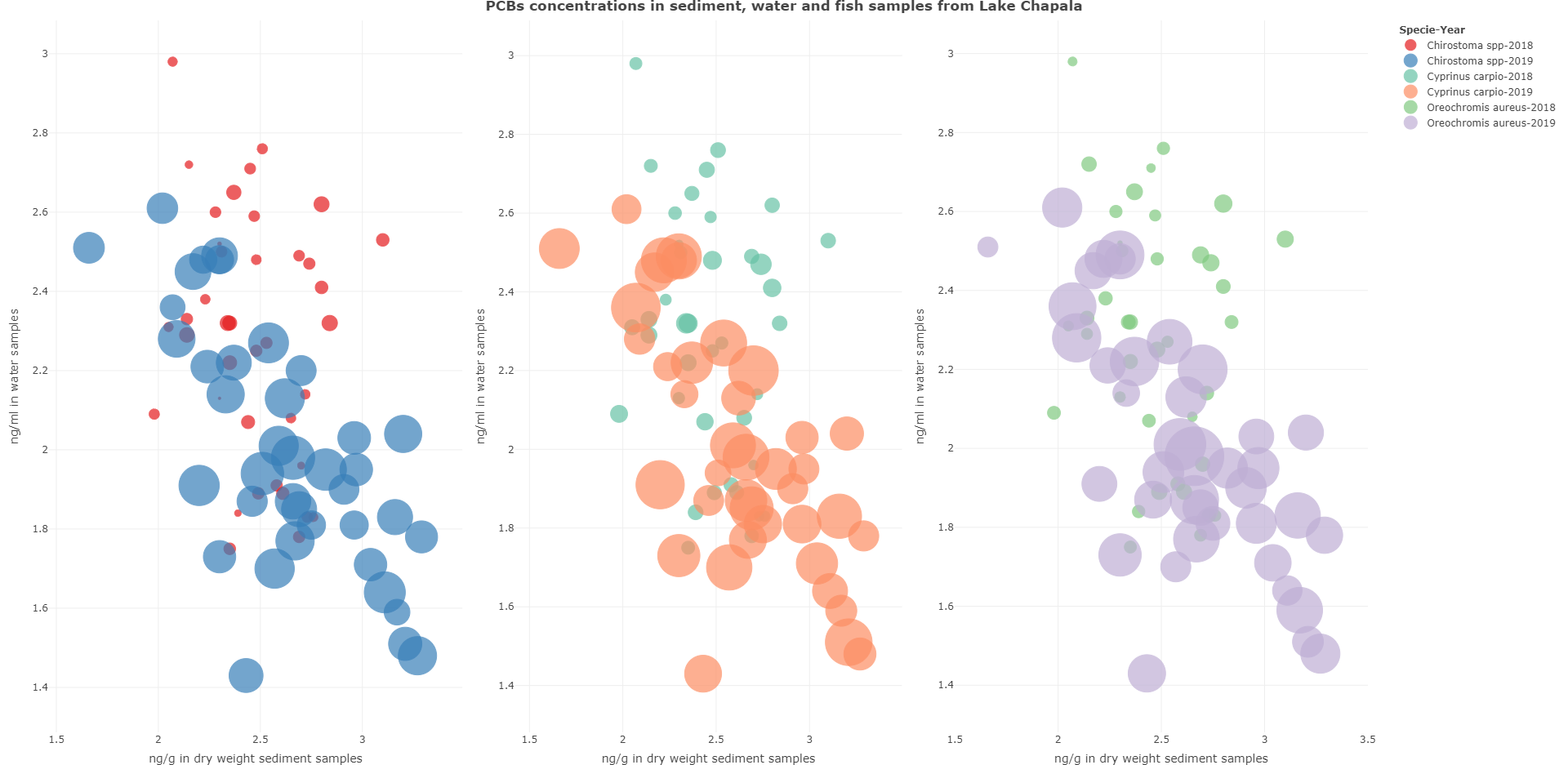

Supplement: Supplementary file 1 [file toxics-09-00241-s001.zip › Figure S1.png]

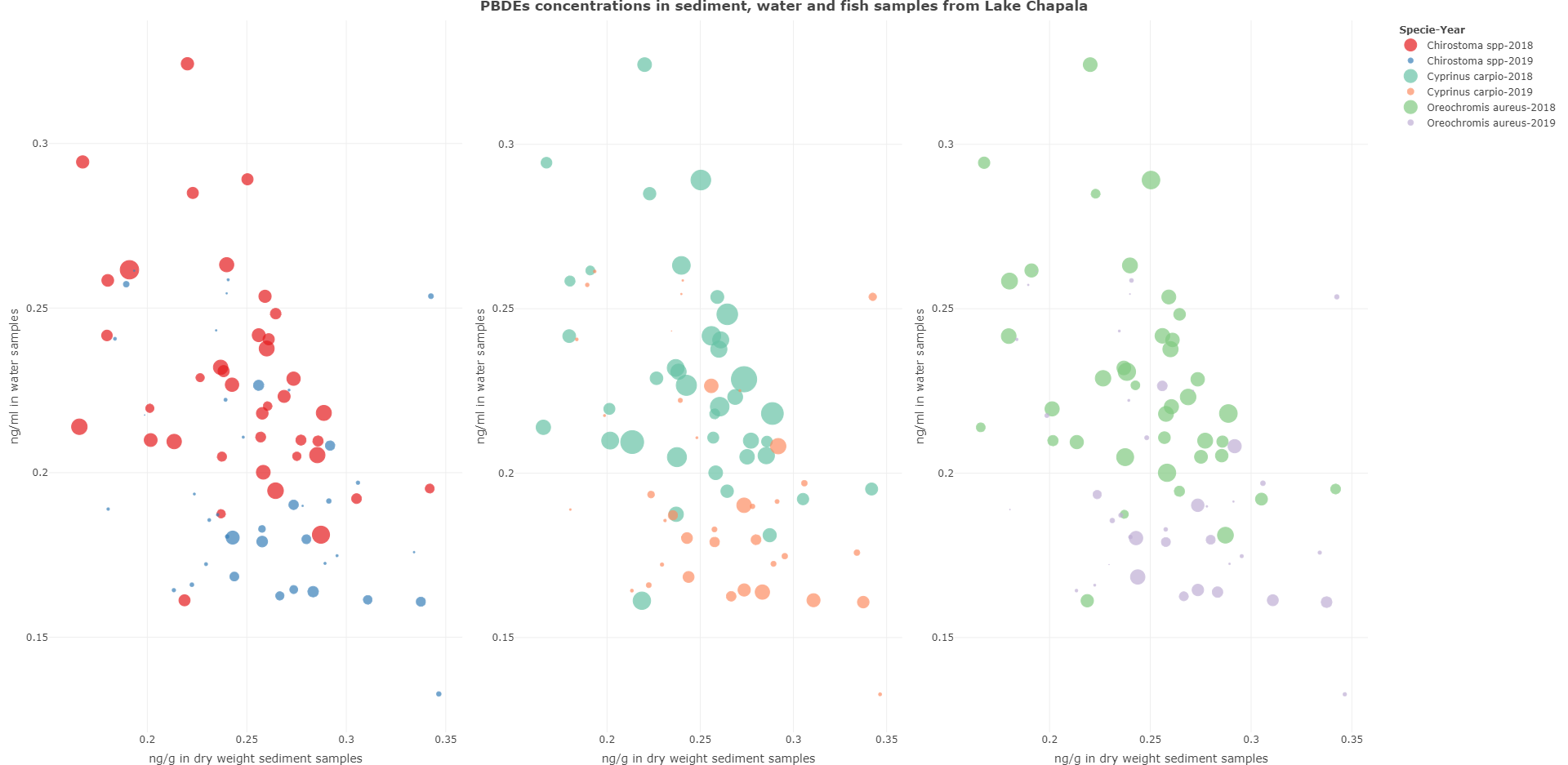

Supplement: Supplementary file 1 [file toxics-09-00241-s001.zip › Figure S2.png]
